# Neutropenic Enterocolitis in a Metastatic Seminoma Patient With Streptococcus gallolyticus Bacteremia

**DOI:** 10.7759/cureus.54077

**Published:** 2024-02-12

**Authors:** Sona Trika, Nicholas R Munoz, Mueez Hussain, Youstina Beshay-Taylor, Zainub Javed

**Affiliations:** 1 Internal Medicine, Western University of Health Sciences College of Osteopathic Medicine of the Pacific, Pomona, USA; 2 Internal Medicine, Southwest Healthcare, Temecula, USA; 3 Internal Medicine, Services Institute of Medical Sciences (SIMS), Lahore, PAK

**Keywords:** high fever, hematology disorders, clinical infectious disease, paralytic ileus, streptococcus gallolyticus, infections in neutropenic fever, oncology patients, testicular seminoma, chemotherapy-induced neutropenia, neutropenic colitis

## Abstract

Neutropenic enterocolitis (NEC), also referred to as typhlitis, is a condition associated with a high mortality risk and primarily manifests in immunocompromised patients. It is characterized by ulceration, edema, and hemorrhage affecting the bowel wall. The underlying cause of NEC is postulated as an immunocompromised condition that facilitates bacterial infiltration through compromised bowel mucosa. The high mortality rate is attributable to bowel necrosis, culminating in perforation and sepsis. This report describes a case involving a patient with metastatic seminoma who exhibited seizure-like activity, fever, *Streptococcus gallolyticus *bacteremia, and NEC. The patient underwent treatment involving broad-spectrum antibiotics and filgrastim. The patient's neutropenia resolved leading to discharge on oral antibiotics. The case reported is unique, as it links NEC to *Streptococcus gallolyticus *and seminoma. *Streptococcus gallolyticus *has not been previously associated with NEC.

## Introduction

Neutropenic enterocolitis (NEC) can result from the usage of chemotherapy drugs, hematopoietic stem cell transplant, corticosteroids, nutritional deficiencies, and severe infections. In the context of chemotherapy-induced NEC, true incidence rates are unknown but are estimated to range between 1% and 7% of patients, but estimates vary based on factors, including the specific patient population and the intensity of chemotherapy. For example, age, cancer type, treatment history, and overall health status can affect incidence rate estimates. Specific drugs, including high-dose cytarabine, fludarabine, and anthracyclines, are most commonly implicated in NEC. Mucosal injury, often induced by chemotherapy, followed by bacterial superinfection, can lead to bacteremia, neutropenia, and neutropenic fever [1].

Bloodstream infections represent one of the most common complications in neutropenic cancer patients, and if attributed to gram-negative rods, are associated with a high mortality rate [2]. Additionally, these patients are susceptible to infections caused by *Clostridium difficile*, Cytomegalovirus (CMV), and strongyloidiasis [3]. Neutropenia is usually defined as absolute neutrophil count (ANC) <1500 cells/uL. Fever during neutropenia warrants empiric antibiotic therapy with proper coverage, as antimicrobial resistance is of great concern in immunocompromised cancer patients.

Management of NEC is often a combination of supportive therapy (bowel rest with nasogastric suction, intravenous fluids, parenteral nutrition) and antibiotic treatment according to exposure and microbial resistance pattern. Empiric therapy should cover gram-negative bacteria and anaerobic organisms that commonly cause NEC. Antibiotics include piperacillin-tazobactam, a carbapenem, or an antipseudomonal cephalosporin in combination with metronidazole. In patients with mucositis, treatment directed against gram-positive bacteria should be considered [4,5].

While NEC is commonly identified as a complication of chemotherapy, there are no reports of patients with seminoma developing NEC with concordant gastrointestinal ileus and *Streptococcus gallolyticus* bacteremia in the context of bleomycin, etoposide, cisplatin (BEP) therapy (a common chemotherapy regimen in seminoma patients). This article highlights the case of a 59-year-old male who developed these complications two weeks after receiving a single dose of BEP therapy.

## Case presentation

A 59-year-old-male with a past medical history of stage four seminoma (with metastasis to the lungs), orchiectomy, erectile dysfunction, hypertension, and hyperlipidemia presented to the emergency department with severe abdominal pain, abdominal bloating, generalized weakness, and seizure-like activity. The patient endorsed fatigue starting in the morning, which he attributed to a recent chemotherapy session. Later in the day, he was resting in his chair when he became unresponsive. His family witnessed his eyes roll back and arms tense up, followed by full-body jerking, and urinary incontinence. The family stated the seizure-like activity lasted under a minute and was followed by brief postictal confusion. Additionally, the patient complained of abdominal cramping and constipation, stating he had not had a bowel movement for over 36 hours.

The patient explained he was initially diagnosed with seminoma eight years previously. At that time, he received an orchiectomy followed by two cycles of adjuvant carboplatin therapy. Two months before presentation to the emergency department (ED), he noticed an enlarging neck mass. He subsequently received a computed tomography (CT) scan showing multiple pulmonary nodules and retroperitoneal lymphadenopathy. He received a biopsy of one of the lung nodules, which revealed CD30+ metastatic seminoma. He was diagnosed with recurrent metastatic seminoma, with plans to receive four cycles of BEP therapy. The patient received his first dose of chemotherapy two weeks prior to presentation. He was given 30 units of intravenous (IV) bleomycin, 100 mg/m^2^ of IV etoposide, 20 mg/m^2^ of IV cisplatin, and 125 mg of IV methylprednisolone. His second dose was scheduled for one month after the first dose.

**Figure 1:**
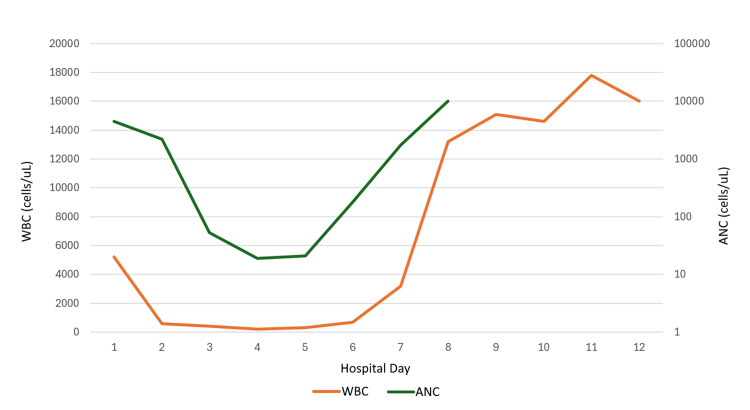
Graphical representation of the patient's WBC count and ANC trend during his hospitalization The patient's WBC trend is shown in orange, and the patient's ANC count is shown in green. His WBC count and ANC were at their lowest point on hospital day four at 200 cells/uL, and 19 cells/uL respectively. Filgrastim was started on hospital day four. ANC: absolute neutrophil count; WBC: white blood cell count

The patient presented to the ED with a blood pressure of 114/73 mmHg, a respiratory rate of 20 breaths per minute, and a heart rate of 83 beats per minute. He was initially afebrile with a temperature of 36.8 °C, although he developed a fever that peaked at 38.7 °C. On physical exam, his heart had a regular rate and rhythm, his lungs were clear to auscultation bilaterally, and his abdomen was distended with diffuse tenderness to palpation. His labs showed a hemoglobin level of 12.0 g/dL, platelets of 291,000 platelets/uL, sodium of 135 mmol/L, blood urea nitrogen (BUN) of 27 mg/dL, creatinine of 1.4 mg/dL, lactic acid of 1.6 mmol/L, normal liver function enzymes, and a procalcitonin of 2.770 ng/mL (Table 1). His white blood cell (WBC) count was initially normal at 5,200 cells/uL, but declined during his hospitalization, reaching a nadir of 200 cells/uL on day four. Additionally, his ANC was initially normal at 4,435 cells/uL, declining to a nadir of 19 cells/uL on hospital day four (Figure 1).

**Table 1:** The patient's lab values on admission BUN: blood urea nitrogen; AST: aspartate aminotransferase; ALT: alanine transaminase

	Patient's Lab Values
Hemoglobin	12.0 g/dL
Platelets	291,000 platelets/uL
Sodium	135 mmol/L
BUN	27 mg/dL
Creatinine	1.4 mg/dL
Procalcitonin	2.770 ng/mL
Alkaline Phosphate	99 IU/L
AST	21 IU/L
ALT	26 IU/L
Total Bilirubin	0.4 mg/dL
Lactic Acid Level	1.6 mmol/L

**Figure 2:**
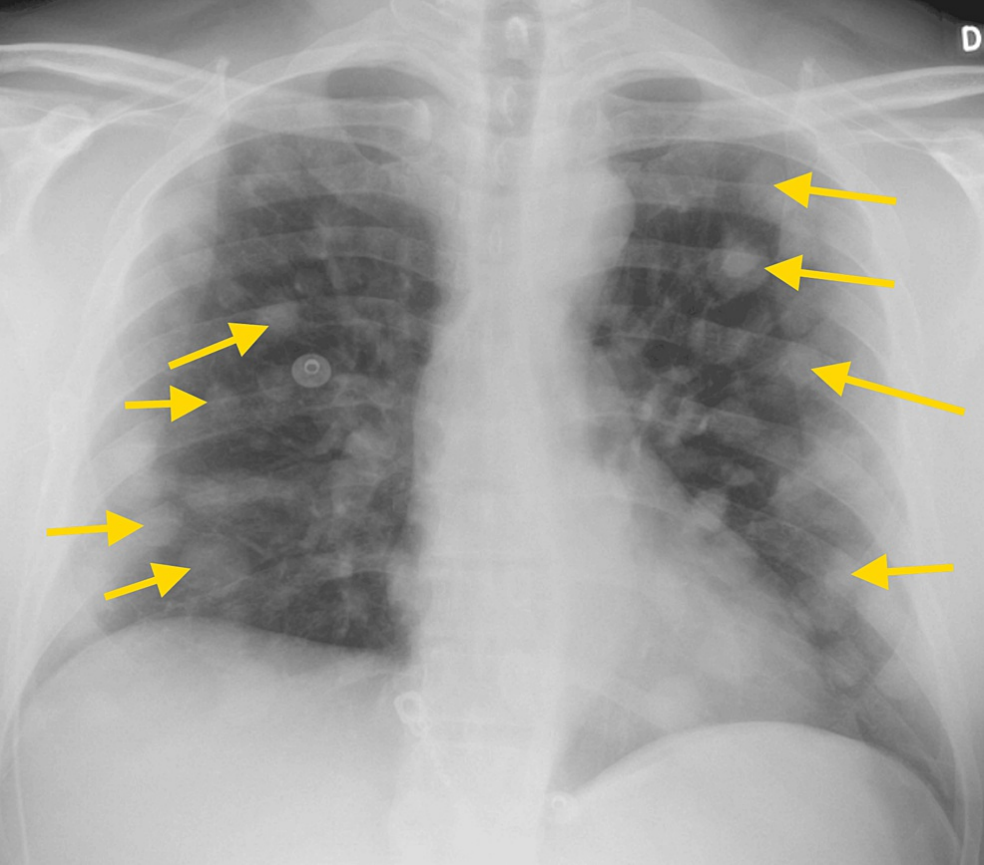
AP chest X-ray of the patient showing numerous pulmonary nodules Several of the pulmonary nodules are pointed out with yellow arrows. AP: anteroposterior

**Figure 3:**
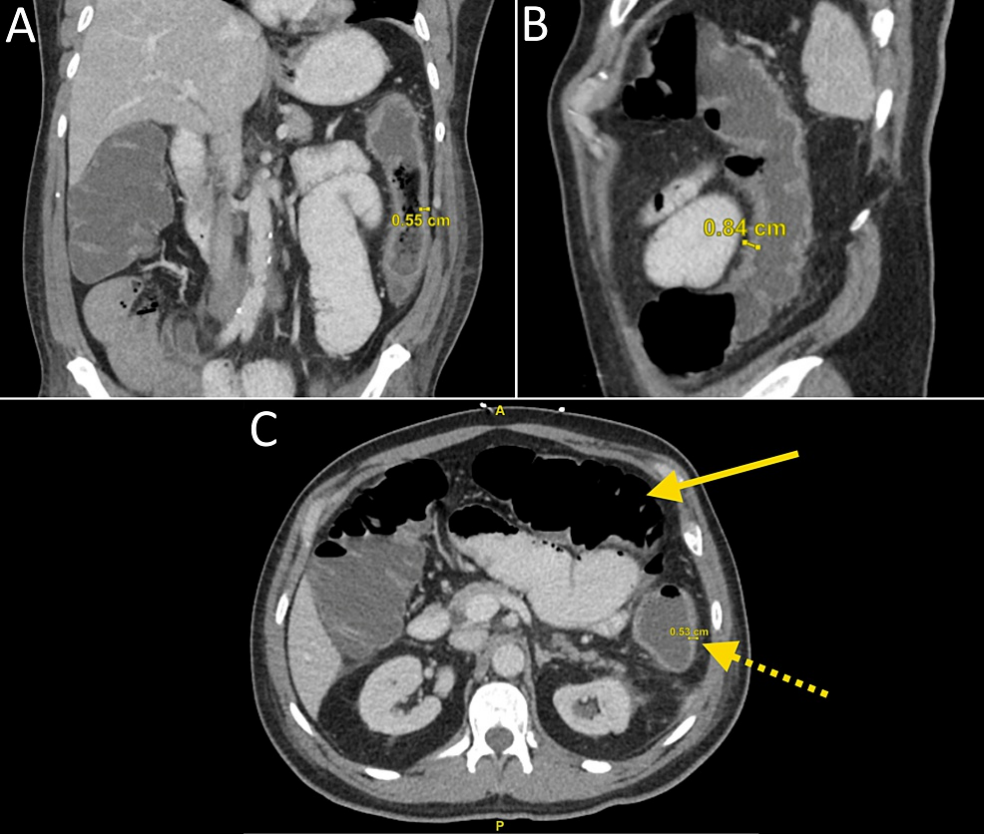
A CT scan of the patient's abdomen and pelvis with contrast showing: (A) coronal view of the descending colon with a wall thickness measuring 5.5 mm, (B) sagittal view of the descending colon with a wall thickness measuring 8.4 mm, (C) transverse view of the descending colon with a wall thickness measuring 5.3 mm as indicated by a dotted arrow and dilation of the small bowel as indicated by a solid arrow CT: computed tomography

The patient's chest X-ray on admission demonstrated numerous lung nodules measuring one to two centimeters (Figure 2). He received magnetic resonance imaging of the brain that showed no abnormalities. On his second day of hospitalization, the patient received blood cultures that were positive for *Streptococcus gallolyticus*. Stool culture was negative for Escherichia coli 0157, salmonella, shigella, or campylobacter. Due to increasing abdominal distention and pain, the patient received a CT scan of the abdomen showing small bowel dilatation likely representing ileus, with thickening of the descending colon measuring up to 8.4 mm (Figure 3). The patient was placed on empiric treatment with piperacillin-tazobactam 4.5 g every eight hours. Later culture sensitivities showed the bacteria was sensitive to the antibiotic. One day after the initiation of IV antibiotics, the patient received another set of blood cultures, which were negative for bacteremia. He received a transesophageal echocardiogram demonstrating no valvular vegetations, ruling out infective endocarditis. The infectious disease team was consulted, recommending starting filgrastim 300 ug daily until the ANC rose to above 500 cells/uL. They recommended that piperacillin-tazobactam be given until the ANC was greater than 1,000 cells/uL. On hospital day seven, the patient’s ANC rose to 1,728 cells/uL; subsequently, filgrastim and piperacillin-tazobactam were discontinued. After discontinuation of IV antibiotics, a 14-day course of oral levofloxacin 750 mg daily and amoxicillin-clavulanate 875-125 mg twice daily was initiated. When the patient's fever and neutropenia resolved, he was discharged with instructions to follow up with his oncologist and a gastroenterologist for a future colonoscopy. After discharge, his oncologist started him on low-dose prophylactic filgrastim to prevent recurrent neutropenia, followed by combination chemotherapy two weeks later.

## Discussion

Symptoms of NEC include abdominal pain, cramping, distention, nausea, vomiting, diarrhea, hematochezia, and fever. Since it can mimic several other diagnoses, including ischemic colitis, appendicitis, and inflammatory bowel disease, it can be difficult to diagnose even when providers are paying close attention to laboratory and radiographic findings. NEC should be suspected in patients who have recently received chemotherapy and present with severe neutropenia (ANC < 500 cells/uL). Often, it is present in those being treated for acute leukemia and less frequently in those with solid tumors (5% incidence). It can develop in patients with primary bone marrow failure unrelated to chemotherapy [6]. Initial labs should include a complete blood count with a differential to assess for neutropenia. The preferred imaging modality in NEC is a CT scan with oral and IV contrast. The most common finding on the CT scan is bowel wall thickening of > 4 mm [7]. Other findings include bowel dilation, wall nodularity, mesenteric stranding, mucosal enhancement, and pneumatosis [8]. Suggested diagnostic criteria vary but the triad of fever, neutropenia, abdominal pain, and bowel wall thickening > 4 mm is well-recognized as appropriate for NEC [9]. *Clostridium difficile* should be ruled out due to the similarity in presentation. Barium enema and colonoscopy are both contraindicated in NEC due to the risk of perforation and resulting bacteremia [1].

The management of NEC should be individualized based on the needs of the patient. In the presence of intestinal perforation with intra-abdominal free air, uncontrollable gastrointestinal bleeding, or peritonitis, surgical intervention is recommended [10]. Due to the high risk of surgery in neutropenic patients, it should be avoided if possible. Medical management includes bowel rest, blood transfusions as needed, parenteral nutrition, abdominal decompression with a nasogastric tube, and antibiotics [11]. Head-to-head trials evaluating the effectiveness of different antibiotic regimens to treat NEC are lacking. Antibiotics should be broad targeting anaerobic, gram-positive, and gram-negative bacteria, including *Pseudomonas aeruginosa*. Monotherapies such as piperacillin-tazobactam or dual therapies, such as cefepime and metronidazole, are effective. The duration of antibiotics for NEC should be continued for, at the minimum, the amount of time the patient is neutropenic. Antibiotic therapy should be lengthened if perforation, bacteremia, or abscess is present. There are no trials analyzing the use of recombinant granulocyte-colony stimulating factor (G-CSF), such as filgrastim, in NEC. Although the benefit of recombinant G-CSF has not been proven, it may be beneficial in patients with extreme neutropenia (such as ANC <100 cells/uL), hypotension, sepsis, or fungal infection [12].

*Streptococcus (S.) gallolyticus* belongs to a group of bacteria known as *Streptococcus bovis/Streptococcus equinus complex* (SBSEC), which was previously known as Group D streptococci. SBSEC includes four major species: *S. gallolyticus*, *S. lutetiensis*, *S. infantarius*, and *S. pasteurianus* [13]. *Streptococcus gallolyticus* has been associated with the rumen of herbivores; less commonly, it has been found in the flora of the human gastrointestinal tract (2.5-15% of people). *Streptococcus gallolyticus* is a major agent that causes endocarditis and septicemia in immunocompromised patients [14]. It has been associated with colorectal cancer. One study found in patients with *Streptococcus gallolyticus* infection who underwent colonoscopy, 60% were found to have concomitant carcinomas or adenomas [15]. Bacteria associated with NEC include *Escherichia coli*, *Pseudomonas aeruginosa*, viridans group streptococci, Klebsiella species, enterococci, Bacteroides species, Clostridium species, and fungi such as Candida [7]. *Streptococcus gallolyticus* has not been previously associated with NEC. Patients with *Streptococcus gallolyticus* bacteremia should undergo an echocardiogram, colonoscopy, and workup for underlying liver disease (such as liver function and viral hepatitis testing) [16].

Interestingly, although it has co-occurred in cases of NEC, ileus is not commonly identified as a complication of BEP chemotherapy treatment. Mechanical ileus is an occlusion or paralysis of the bowel that prevents the forward passage of intestinal contents. Ileus can be due to external compression, inflammation, or lumenal blockage. In contrast, functional ileus can be drug-induced, metabolic, vascular, or refractory to surgery. Drugs that impair bowel motility, such as opioids, anticholinergics, calcium channel blockers, antidepressants, and antipsychotics, are implicated in causing functional ileus. The diagnosis of ileus is dependent on radiographic evidence, including a CT scan or small bowel series. A metabolic panel is essential to identify potential reversible factors contributing to ileus such as hypokalemia. A comprehensive blood count is necessary to rule out complications such as bleeding or infection [17].

The patient presented likely developed ileus before the onset of NEC, as he was experiencing worsening constipation and abdominal pain in the days prior to hospitalization. The patient did not receive testing for *Clostridium difficile*, as he did not have ≥3 loose stools in 24 hours. The BEP chemotherapy the patient received two weeks before hospitalization likely led to an immunocompromised state, ileus, and a compromised bowel wall mucosa. The initially normal WBC count followed by neutropenia was likely due to early presentation in the patient's disease course. *Streptococcus gallolyticus* bacteria from the patient's intestine likely translocated through the weakened wall of the colon, leading to neutropenia, fever, positive blood cultures, and bowel wall thickening. The patient's fever, neutropenia, and bacteremia resolved with appropriate treatment. He was discharged with instructions to follow up with his oncologist and a gastroenterologist for a future colonoscopy. 

## Conclusions

The case is a unique presentation of NEC in the context of *Streptococcus gallolyticus* bacteremia. It illuminates the intricate interplay between severe neutropenia, compromised intestinal integrity, and bacterial translocation. It highlights the importance of early recognition and intervention in NEC, particularly within the context of aggressive chemotherapy regimens. Notably, the patient's *Streptococcus gallolyticus* bacteremia - typically associated with colorectal carcinoma - illustrates the diverse and unpredictable microbial challenges in neutropenic patients. The case highlights the need for personalized treatment strategies tailored to the patient’s presentation and hospital course. It underscores the critical role of multidisciplinary care. Ongoing research and clinical vigilance remain essential for optimizing outcomes in patients at risk for NEC.
